# Determinants and prognostic value of atrial and ventricular ectopic burden in adults without structural heart disease

**DOI:** 10.1093/ehjopen/oeag133

**Published:** 2026-08-30

**Authors:** Amit Moses, Rotem Givoli Vilensky, Noam Makmal, Kobi Faierstein, Elad Maor, Roy Beinart, Avi Sabbag

**Affiliations:** Leviev Heart Center, Sheba Medical Center, Affiliated with Sackler School of Medicine, Tel Aviv University, Sheba Road 2, Ramat Gan, Tel Aviv 5265601, Israel; Internal Medicine Ward C, Sheba Medical Center, Affiliated with Sackler School of Medicine, Tel Aviv University, Sheba Road 2, Ramat Gan, Tel Aviv 5265601, Israel; Leviev Heart Center, Sheba Medical Center, Affiliated with Sackler School of Medicine, Tel Aviv University, Sheba Road 2, Ramat Gan, Tel Aviv 5265601, Israel; Leviev Heart Center, Sheba Medical Center, Affiliated with Sackler School of Medicine, Tel Aviv University, Sheba Road 2, Ramat Gan, Tel Aviv 5265601, Israel; Leviev Heart Center, Sheba Medical Center, Affiliated with Sackler School of Medicine, Tel Aviv University, Sheba Road 2, Ramat Gan, Tel Aviv 5265601, Israel; Leviev Heart Center, Sheba Medical Center, Affiliated with Sackler School of Medicine, Tel Aviv University, Sheba Road 2, Ramat Gan, Tel Aviv 5265601, Israel; Davidai Arrhythmia Center, Sheba Medical Center, Affiliated with Sackler School of Medicine, Tel Aviv University, Sheba Road 2, Ramat Gan, Tel Aviv 5265601, Israel; Leviev Heart Center, Sheba Medical Center, Affiliated with Sackler School of Medicine, Tel Aviv University, Sheba Road 2, Ramat Gan, Tel Aviv 5265601, Israel; Davidai Arrhythmia Center, Sheba Medical Center, Affiliated with Sackler School of Medicine, Tel Aviv University, Sheba Road 2, Ramat Gan, Tel Aviv 5265601, Israel

**Keywords:** Atrial ectopy, Ventricular ectopy, Holter monitoring, Cardiovascular outcomes, Risk stratification, Primary prevention

## Abstract

**Aims:**

Atrial and ventricular ectopy are frequently encountered during ambulatory monitoring in asymptomatic adults; however, their independent long-term prognostic significance in low-risk populations without structural heart disease remains a subject of ongoing clinical debate.

**Purpose:**

To identify the distinct clinical and physiological determinants of atrial ectopic burden (AEB) and ventricular ectopic burden (VEB) and to evaluate their independent long-term associations with major adverse cardiovascular events (MACE) in a primary prevention cohort.

**Methods and results:**

We evaluated 931 asymptomatic individuals (mean age 52 ± 7 years; 13% female) without structural heart disease who completed a baseline 24-h Holter recording. High AEB and VEB were defined using cohort-specific medians and/or the presence of ambulatory arrhythmic complexity. Complete longitudinal tracking for MACE (composite of acute coronary syndrome, cerebrovascular accident, new-onset heart failure, or cardiovascular mortality) was available for 840 participants over a median follow-up of 8 years (IQR 5–11 years). To evaluate the robustness of the atrial findings, a prespecified sensitivity analysis was performed by excluding patients with baseline atrial fibrillation. Over the follow-up period, the primary MACE endpoint occurred in 149 (18%) participants. In multivariable logistic regression models, high AEB was independently driven by older age (OR 1.09, 95% CI 1.06–1.12; *P* < 0.001), male sex (OR 1.60, 95% CI 1.02–2.52; *P* = 0.042), reduced estimated glomerular filtration rate (eGFR) (OR 0.98, 95% CI 0.97–0.99; *P* = 0.049), and low cardiorespiratory fitness (OR 1.38, 95% CI 1.01–1.92; *P* = 0.049). Conversely, high VEB was independently associated only with older age (OR 1.04, 95% CI 1.02–1.07; *P* < 0.001) and reduced eGFR (OR 0.98, 95% CI 0.977–0.99; *P* = 0.034). In fully adjusted multivariable Cox proportional hazards models, high VEB remained a robust independent predictor of MACE, conferring a three-fold increase in risk (HR 3.06, 95% CI 2.06–4.56; *P* < 0.001). In tertile-based analyses of pure ectopic burden, increasing VEB demonstrated a robust graded association with MACE after adjustment for age, sex, beta-blocker use, and antiarrhythmic drug therapy (HR per tertile increase 2.02, 95% CI 1.60–2.53; *P* < 0.001), whereas AEB showed only a borderline association that did not reach statistical significance (HR per tertile increase 1.21, 95% CI 0.98–1.50; *P* = 0.076).

**Conclusion:**

In asymptomatic adults without structural heart disease, the clinical determinants and long-term prognostic trajectories of ectopy exhibit a profound chamber-specific divergence. Ventricular ectopic burden stands out as a powerful, independent marker of intrinsic electrical vulnerability and long-term cardiovascular risk. Meanwhile, atrial ectopy is highly sensitive to systemic physiological fitness and carries a weaker prognostic signal that attenuates completely upon multivariate clinical and medication adjustment.

## Introduction

Atrial and ventricular ectopic beats are frequently identified during ambulatory electrocardiographic monitoring and have traditionally been regarded as incidental, benign findings in individuals with structurally normal hearts. While these ectopic beats are common in asymptomatic adults and exhibit a progressive increase with age, their clinical significance remains a subject of debate. Emerging epidemiological evidence has begun to challenge the assumption that such ectopy is harmless, linking even a modest ectopic burden to an increased risk of atrial fibrillation, stroke, heart failure, and increased mortality.^1–3^ The persisting discrepancy between routine clinical reassurance and these emerging risk signals creates significant uncertainty regarding the interpretation of ectopy in preventive cardiology.^1–7^ Much of the existing literature has focused on older or high-risk populations. In such cohorts, the presence of significant comorbidities and underlying structural heart disease often obscures the independent contribution of ectopic activity to cardiovascular outcomes. Consequently, there is a notable lack of robust data concerning the prevalence, burden, and long-term prognostic relevance of ectopy in adults without established cardiovascular disease.^8–11^

To address this gap, we aimed to characterize atrial and ventricular ectopic burden and evaluate its clinical relevance in a large cohort of asymptomatic adults. By utilizing systematic assessment at a tertiary prevention centre, this study seeks to determine whether disproportionate ectopic activity even at low absolute burdens serves as an early biomarker of electrical vulnerability and a predictor of long-term cardiovascular events.

## Materials and methods

The population of the Institute for Medical Screening at the Chaim Sheba Medical Center has been described previously.^12^ Briefly, the Institute performs routine annual examinations of asymptomatic healthy adults. The yearly basic evaluation includes an examination by a physician, a detailed medical history, routine blood work, and exercise stress tests. Subjects were referred for further evaluation based on their symptoms or clinical needs. Holter monitoring was performed when clinically indicated.

**Figure 1.**
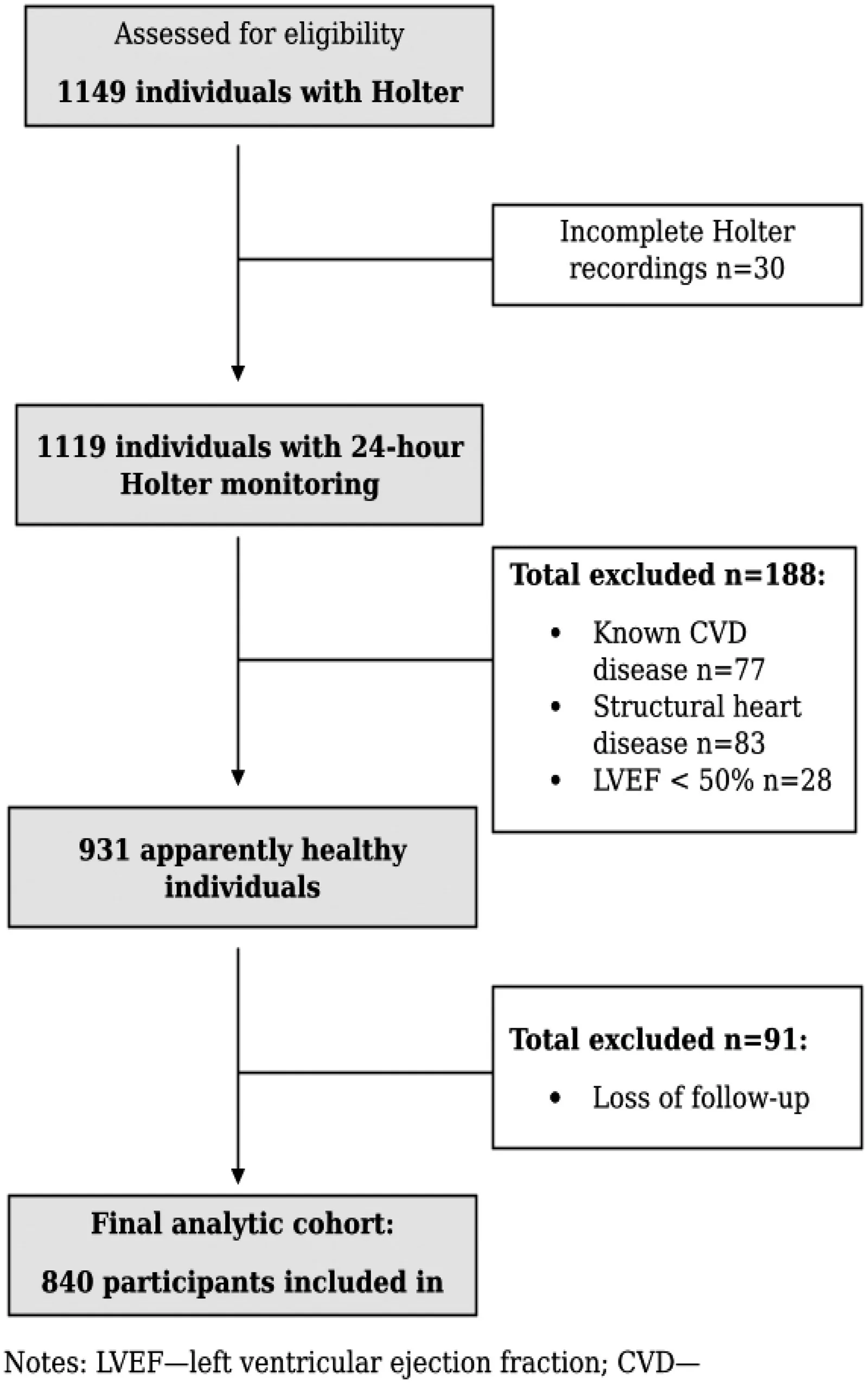
Flow diagram of participant selection and study cohort derivation.

The current study comprised all individuals evaluated at the Sheba survey between 2010 and 2022 who underwent at least a single clinically indicated 24-h Holter monitoring. Individuals were excluded due to incomplete Holter examinations, the presence of structural heart disease, and/or an ejection fraction below 50%. Additional exclusions included a history of acute coronary syndrome (ACS), cerebrovascular accident (CVA), heart failure (HF), prosthetic valve, and pacemaker/ICD implantation (*Figure 1*). Baseline demographics, cardiovascular history, clinical risk factors, laboratory data, and medication exposure, including beta-blockers and antiarrhythmic drugs primarily comprising Class IC agents (e.g. flecainide, propafenone) and Class III agents (e.g. dronedarone), were extracted from institutional electronic medical records. The study protocol was approved by the institutional ethics committee and conducted in accordance with the Declaration of Helsinki; the requirement for informed consent was waived.

When multiple Holter recordings were performed, only the earliest was considered. Holter recordings were evaluated by trained electrophysiologists in accordance with contemporary European Society of Cardiology standards. Atrial ectopic burden (AEB) and ventricular ectopic burden (VEB) were quantified as the percentage of ectopic beats relative to the total number of recorded cardiac cycles. Because conventional absolute thresholds established for higher-risk populations, such as a premature ventricular complex (PVC) burden >5%, were rarely met in this asymptomatic cohort, standard clinical cut-offs precluded meaningful risk stratification. To overcome this limitation and capture subtle electrophysiological vulnerability, we employed a cohort-specific classification. High ectopic burden was defined as an ectopic percentage exceeding the cohort median and/or the presence of arrhythmic complexity, which included atrial couplets, paroxysmal atrial tachycardia (PAT), or atrial fibrillation (AF) for high AEB and ventricular couplets or non-sustained ventricular tachycardia (NSVT) for high VEB.

To evaluate dose-response relationships beyond the prespecified high-burden definitions, pure AEB and VEB percentages were additionally analysed across cohort-specific tertiles. Sensitivity analyses using quartiles and quintiles were also performed to assess the consistency of graded associations between ectopic burden and long-term major adverse cardiovascular events (MACE). Furthermore, because overt baseline AF may represent advanced, established cardiomyopathy rather than isolated ectopic activity, we performed a prespecified sensitivity analysis. For this analysis, all individuals with baseline AF were excluded from the cohort, and the high-AEB exposure threshold was recalculated using the remaining participants to isolate the true prognostic weight of subclinical atrial ectopy.

For individuals undergoing longitudinal evaluation, serial Holter monitoring was defined as the presence of two or more separate recordings on distinct calendar dates. Given that repeat Holter testing was clinically indicated rather than protocol-driven, primary survival analyses were based exclusively on baseline ectopic burden to minimize indication and immortal time biases. Serial Holter data were therefore summarized descriptively by the number and timing of repeat recordings but were not modelled as time-varying exposures. Cardiorespiratory fitness was evaluated via standardized treadmill exercise stress testing and quantified in metabolic equivalents (METs). To account for physiological variations in baseline exercise capacity, fitness was dichotomized into low or high categories utilizing age- and sex-specific thresholds derived from performance quartiles within the cohort.

### Follow-up and study endpoints

The primary outcome was MACE, defined as a composite of the first occurrence of ACS, CVA, new-onset HF, or cardiovascular mortality. Secondary endpoints evaluated the individual components of the primary composite and non-cardiovascular mortality. Time-to-event was calculated from the date of baseline Holter monitoring to the first documented clinical event, last known follow-up, or death. Clinical endpoints were verified through internal institutional electronic medical records and validated via direct linkage with the national death registry.

Statistical analyses were performed using R software (version 4.2.3) and SPSS (version 26). Continuous variables were evaluated for normality and are presented as mean ± standard deviation or median (interquartile range) based on distribution, while categorical variables are expressed as counts and percentages. Baseline characteristics across ectopic burden groups were compared using independent-samples t-tests or Mann–Whitney U tests for continuous variables and χ^2^ or Fisher’s exact tests for categorical variables. Bivariate correlations were assessed using Pearson or Spearman coefficients, depending on data scales and normality.

Multivariable logistic regression was utilized to determine independent clinical and physiological determinants of high atrial and ventricular ectopic burdens. To address potential confounding, expanded logistic regression models were constructed. These fully adjusted models simultaneously integrated baseline demographic characteristics, metabolic factors, and physiological variables, specifically age, sex, body mass index (BMI), smoking status, diabetes mellitus, hypertension, hyperlipidaemia, renal function estimated glomerular filtration rate (eGFR), and cardiorespiratory fitness, to confirm whether these clinical determinants remained independently associated with ectopic activity after controlling for conventional cardiovascular risk factors and comorbidities.

Cumulative event-free survival was estimated using the Kaplan-Meier method, and differences across exposure strata and ectopic burden tertiles were evaluated via the log-rank test. Multivariable Cox proportional hazards regression models were applied for time-to-event analyses, with risk estimates reported as hazard ratios (HR) and 95% confidence intervals (CI). Graded dose-response relationships across ectopic tertiles were evaluated by modelling the categories as ordinal variables in trend tests. Finally, to control for potential confounding by indication due to rate- or rhythm-modulating therapies, baseline usage of beta-blockers and antiarrhythmic drugs was compared between high- and low-burden cohorts. These baseline medication exposures were explicitly forced into the primary multivariable Cox models alongside clinical demographics (age and sex) to calculate fully adjusted hazard ratios. Statistical significance across all models was defined as a two-tailed *P* < 0.05.

## Results

A total of 1149 consecutive individuals who underwent annual preventive evaluations and completed 24-h Holter monitoring were initially screened. This cohort constituted 1.5% of the total population evaluated by the Institute for Medical Screening of the Chaim Sheba Medical Center. As illustrated in the study flow chart (*Figure 1*), we sequentially excluded individuals with incomplete or uninterpretable Holter recordings (*n* = 30), underlying structural heart disease or a baseline left ventricular ejection fraction <50% (*n* = 111), and a history of prior cardiovascular disease (including ACS, CVA, HF, or pacemaker/ICD implantation; *n* = 77). Following these exclusions, the final analytical cohort comprised 931 participants with a mean age of 52 ± 7 years, of whom 13% were female. Regarding baseline cardiovascular risk profiles, hypertension was present in 14% of the total population, obesity (BMI ≥ 30 kg/m^2^) was observed in 15%, and diabetes mellitus was documented in 2% (*Tables 1* and *2*). Baseline echocardiographic parameters are summarized in Supplementary material online, *Table S1*.

**Table 1:** Baseline characteristics of atrial arrhythmias (*n* = 931)

Variable	Low AEB *n* = 370	High AEB *n* = 561	Odds ratio	*P* ^a^	95% CI^b^
Age (years); mean (SD)	49 ± 7	54 ± 7	1.10	<0.001	1.08–1.12
Gender, male; *n* (%)	304 (82%)	505 (90%)	1.96	0.001	1.34–2.88
BMI, ≥30 kg/m^2^; *n* (%)	56 (15%)	81 (14%)	0.95	0.769	0.66–1.37
Hypertension; *n* (%)	37 (10%)	91 (16%)	1.74	0.007	1.17–2.64
Smoking; *n* (%)	41 (11%)	73 (13%)	1.20	0.379	0.80–1.82
LDL (mg/dL); mean (SD)	125 ± 29	126 ± 29	1.00	0.290	—
LDL ≥ 150; *n* (%)	65 (18%)	108 (19%)	1.12	0.518	0.80–1.58
Diabetes (glucose ≥ 126 mg/dL); *n* (%)	7 (1.9%)	9 (1.6%)	0.85	0.741	0.31–2.38
Haemoglobin (mg/dL); mean (SD)	14.72 ± 1.23	14.92 ± 1.09	1.17	0.037	1.04–1.31
CKD-EPI (mL/min/1.73m^2^); mean (SD)	82 ± 13	77 ± 12	0.97	<0.001	0.96–0.98
Low fitness levels (METs); *n* (%)	142 (38%)	151 (27%)	0.65	0.004	0.49–0.88

BMI, body mass index; CKD-EPI, Chronic Kidney Disease Epidemiology Collaboration; CVA, cerebral vascular accident; LDL, low-density lipoprotein cholesterol; METs, metabolic equivalents of task; SD, standard deviation.

^a^ *P*-value < 0.05.

^b^95% Confidence interval.

**Table 2:** Baseline characteristics of ventricular arrhythmias (*n* = 931)

Variable	Low VEB *n* = 429	High VEB *n* = 502	Odds ratio	*P* ^a^	95% CI^b^
Age (years); mean (SD)	51 ± 7	53 ± 7	1.05	<0.001	1.03–1.07
Gender, male; *n* (%)	366 (85%)	443 (88%)	1.29	0.186	0.88–1.89
BMI, ≥30 kg/m^2^; *n* (%)	64 (15%)	73 (15%)	0.97	0.872	0.67–1.40
Hypertension; *n* (%)	54 (13%)	74 (15%)	1.20	0.342	0.83–1.76
Smoking; *n* (%)	48 (11%)	66 (13%)	1.20	0.363	0.81–1.79
LDL (mg/dL); mean (SD)	125 ± 29	126 ± 29	1.00	0.626	—
LDL ≥ 150; *n* (%)	77 (18%)	96 (19%)	1.08	0.646	0.78–1.51
Diabetes (glucose ≥ 126 mg/dL); *n* (%)	8 (1.9%)	8 (1.6%)	0.85	0.751	0.31–2.33
Haemoglobin (mg/dL); mean (SD)	14.77 ± 1.19	14.90 ± 1.11	1.10	0.155	0.98- 1.23
CKD-EPI (mL/min/1.73m^2^); mean (SD)	81 ± 13	78 ± 12	0.98	<0.001	0.97–0.99
Low fitness levels (METs); *n* (%)	214 (50%)	262 (52%)	0.89	0.446	0.67–1.20

BMI, body mass index; CKD-EPI, Chronic Kidney Disease Epidemiology Collaboration; CVA, cerebral vascular accident; LDL, low-density lipoprotein cholesterol; METs, metabolic equivalents of task; SD, standard deviation.

^a^ *P*-value < 0.05.

^b^95% Confidence interval.

Baseline medication profiles varied significantly according to ectopic burden within the follow-up sub-cohort (*n* = 840). Among participants with a high AEB, baseline beta-blocker therapy was more common than in the low-AEB group [167/479 (34.9%) vs. 73/361 (20.2%); *P* < 0.001], as was antiarrhythmic drug utilization [57/479 (11.9%) vs. 11/361 (3.0%); *P* < 0.001]. A parallel distribution pattern was observed across the ventricular cohorts: Individuals with a high VEB demonstrated significantly higher rates of baseline beta-blocker exposure [140/400 (35.0%) vs. 100/440 (22.7%); *P* < 0.001] and antiarrhythmic drug use [49/400 (12.2%) vs. 19/440 (4.3%); *P* < 0.001] compared to their low-burden counterparts.

Baseline ambulatory arrhythmic characteristics are summarized in *Table 3*. The cohort exhibited a median VEB of 0.003% of total beats, with ventricular couplets observed in 14% of participants and NSVT documented in 5%. The median AEB was 0.0026% of total beats; subclinical arrhythmic complexity was common, with atrial couplets present in 32%, PAT in 31%, and baseline AF in 12% of the population (*Table 3*). Across the entire cohort, the mean exercise performance was 12.1 ± 2.3 METs. In multivariable logistic regression models, low cardiorespiratory fitness emerged as a significant independent predictor of a high AEB (OR 1.38, 95% CI 1.01–1.92; *P* = 0.049; *Table 4*). Conversely, exercise capacity demonstrated no independent association with a high VEB in adjusted models (*Table 5*).

**Table 3:** Holter-derived arrhythmic characteristics of the study population

Variable	Overall *n* = 931
Atrial ectopy %; median	0.0026
Ventricular ectopy %; median	0.003
Atrial Couplets; *n* (%)	298 (32%)
Ventricular couplets; *n* (%)	130 (14%)
AF; *n* (%)	115 (12%)
PAT; *n* (%)	289 (31%)
NSVT; *n* (%)	46 (5%)
Mean heart rate (BPM); median	69

AF, atrial fibrillation; PAT, paroxysmal atrial tachycardia; NSVT, non-sustained ventricular tachycardia; BPM, beats per minutes.

**Table 4:** Logistic regression for high atrial ectopic burden (*n* = 931)

Characteristic	OR	95% CI	*P*-value
Age (years)	1.09	1.06, 1.12	<0.001
Gender			
Female			
Male	1.60	1.02, 2.52	0.042
Hypertension	1.17	0.73, 1.90	0.531
BMI ≥ 30 (kg/m^2^)	0.76	0.48, 1.20	0.284
Smoking	1.31	0.82, 2.14	0.264
Diabetes	0.27	0.07, 0.98	0.048
CKD-EPI (mL/min/1.73m^2^)	0.98	0.97, 0.99	0.049
LDL ≥ 150	1.12	0.74, 1.69	0.596
Low fitness levels (METs)	1.45	1.01, 1.92	0.049

BMI, body mass index; CI, confidence interval; CKD-EPI, Chronic Kidney Disease Epidemiology Collaboration; METS, low fitness levels; OR, odds ratio; LDL, low-density lipoprotein cholesterol.

Logistic regression adjusted for age (years), sex, CKD-EPI, hypertension, BMI ≥ 30 (kg/m^2^), smoking, diabetes, LDL ≥ 150, and low fitness levels.

**Table 5:** Logistic regression for high ventricular ectopic burden (*n* = 931)

Characteristic	OR	95% CI	*P*-value
Age (years)	1.04	1.02, 1.07	<0.001
Gender			
Female			
Male	1.13	0.76, 1.68	0.545
Hypertension	0.94	0.63,1.40	0.745
BMI ≥ 30 (kg/m^2^)	0.96	0.66, 1.41	0.830
Smoking	1.31	0.88,1.97	0.193
Diabetes	0.59	0.21, 1.65	0.302
CKD-EPI (mL/min/1.73m^2^)	0.98	0.97, 0.99	0.034
LDL ≥ 150	1.08	0.76,1.52	0.674

BMI, body mass index; CI, confidence interval; CKD-EPI, Chronic Kidney Disease Epidemiology Collaboration; METS, low fitness levels; OR, odds ratio; LDL, low density lipoprotein cholesterol.

Logistic regression adjusted for age (years), sex, CKD-EPI, hypertension, BMI ≥ 30 (kg/m^2^), smoking, diabetes, and LDL ≥ 150

### Clinical determinants of atrial ectopic burden

In the expanded multivariable logistic regression model (*Table 4*), older age remained a powerful independent determinant of high AEB (OR 1.09, 95% CI 1.06–1.12; *P* < 0.001). Male sex was associated with a 60% increase in the odds of exhibiting a high atrial burden (OR 1.60, 95% CI 1.02–2.52; *P* = 0.042). Additionally, diminished renal function (OR 0.98 per unit decrease in eGFR, 95% CI 0.97–0.99; *P* = 0.049) and low cardiorespiratory fitness (OR 1.38, 95% CI 1.01–1.92; *P* = 0.049) were identified as significant independent predictors of advanced atrial ectopy. Other traditional metabolic risk factors showed no independent association with high AEB.

### Clinical determinants of ventricular ectopic burden

In the parallel multivariable logistic regression model adjusted for the same clinical covariates (*Table 5*), older age was independently associated with an increased risk of high VEB, though with a lower effect size than observed in the atrium (OR 1.04, 95% CI 1.02–1.07; *P* < 0.001). Lower renal function also remained a significant predictor of ventricular burden (OR 0.98, 95% CI 0.97–0.99; *P* = 0.034). In stark contrast to the atrial findings, male sex, low cardiorespiratory fitness, and standard metabolic risk factors demonstrated no independent statistical association with a high VEB.

**Figure 2:**
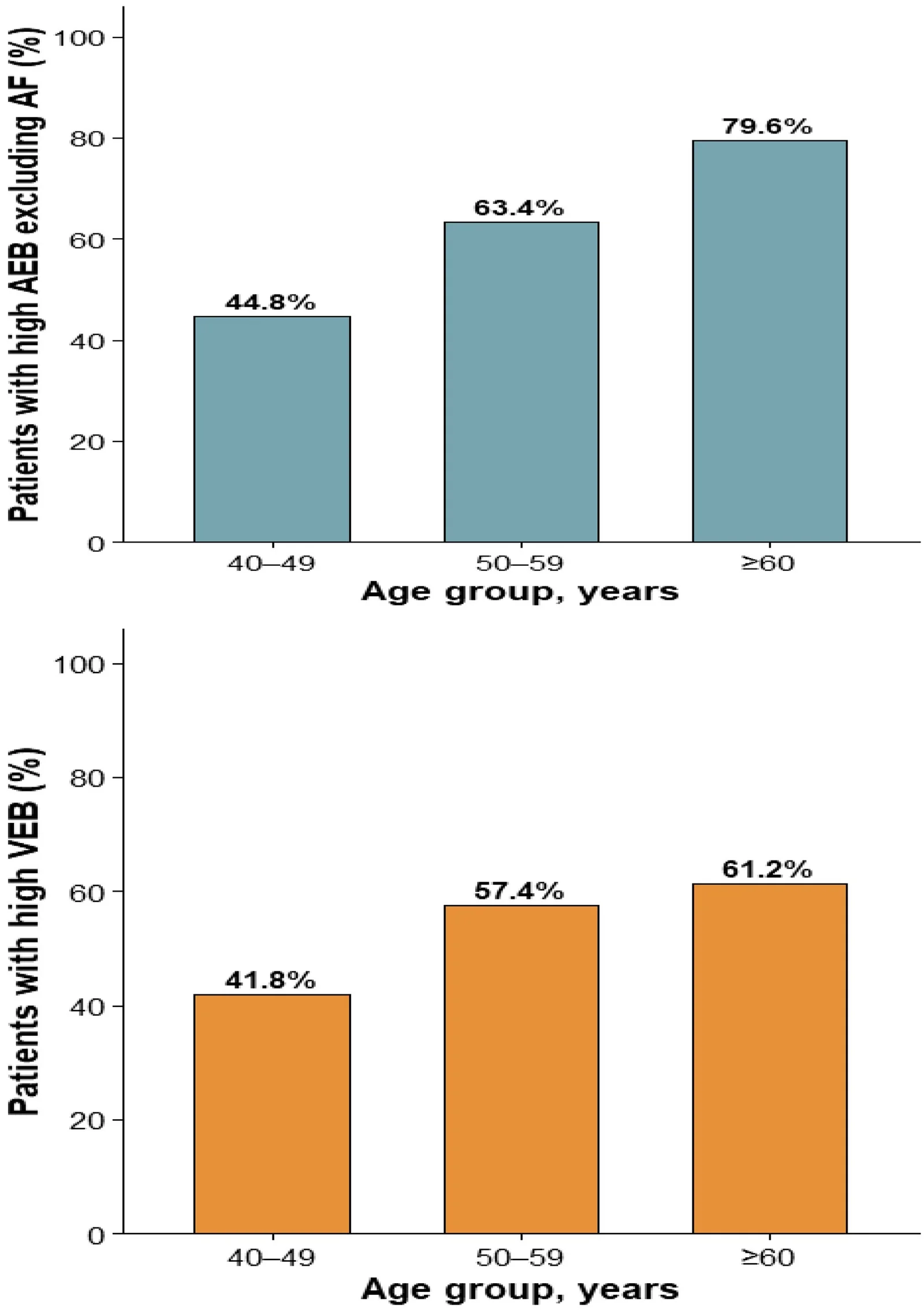
Prevalence of high atrial and ventricular ectopic burden according to age group. Bars represent the proportion of participants in each age group meeting the prespecified criteria for high AEB or high VEB. High AEB was defined as supraventricular ectopic burden above the cohort median and/or supraventricular couplets and/or PAT, excluding AF from the definition. High VEB was defined as ventricular ectopic burden above the cohort median and/or ventricular couplets and/or NSVT.

Ectopic burden increased progressively with advancing age, whether evaluated as a continuous variable or categorized by the prespecified high-burden definitions. Due to the highly right-skewed distribution and low absolute values of continuous ectopy metrics, the data are graphically represented as the proportion of participants within each age stratum meeting the high-burden criteria (*Figure 2*). Specifically, the prevalence of high AEB (excluding AF) increased from 44.8% in individuals aged 40–49 years to 63.4% in those aged 50–59 years, reaching 79.6% in the ≥60 years cohort. A parallel age-dependent escalation was observed for high VEB, rising from 41.8% to 57.4% and 61.2% across the respective age groups.

In the fully adjusted, expanded multivariable logistic regression model (*Table 5*), older age (OR 1.04, 95% CI 1.02–1.07; *P* < 0.001) and lower renal function (OR 0.98, 95% CI 0.97–0.99; *P* = 0.034) were confirmed as the only independent clinical determinants of a high VEB. Conversely, cardiorespiratory fitness demonstrated no independent statistical relationship with ventricular ectopy (*P* = 0.70), and no independent associations were observed for traditional cardiovascular and metabolic risk factors (*Table 5*).

### Survival outcomes

**Figure 3:**
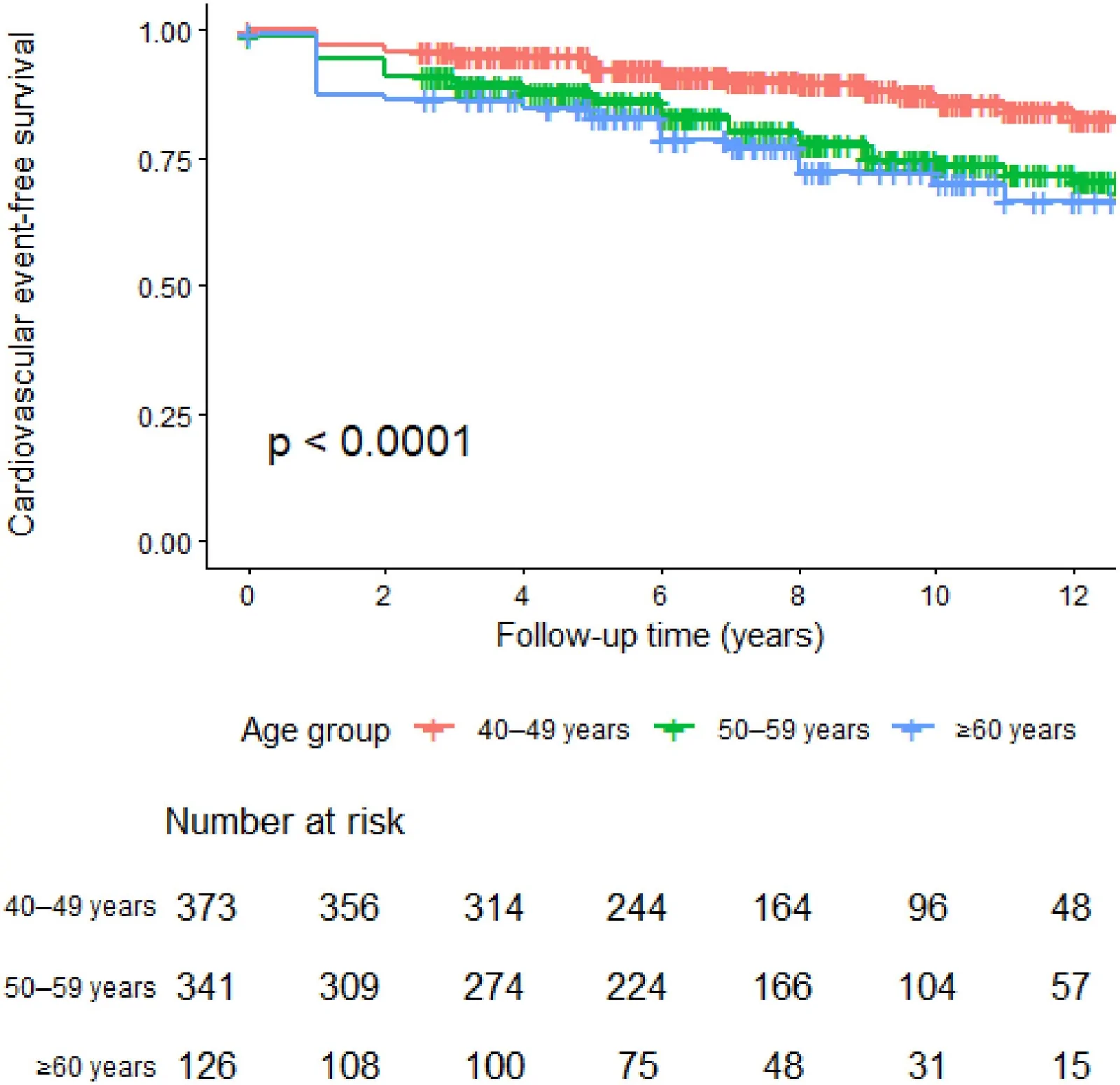
Kaplan-Meier estimates of cardiovascular event-free survival among participants aged 40–49, 50–59, and ≥60 years. Event-free survival differed significantly across age groups by log-rank test (*P* < 0.0001).

Longitudinal follow-up data were complete for 840 participants, spanning a median tracking duration of 8 years (interquartile range [IQR]: 5–11 years; *Table 6*). During this period, 149 individuals experienced a first MACE, with the cumulative event incidence encompassing 70 cases of ACS, 57 CVA, and 29 cases of new-onset HF. All-cause mortality occurred in 15 participants (1.8%), with the vast majority driving non-cardiovascular endpoints; only a single cardiovascular death was recorded, which was attributed to terminal heart failure (*Table 6*). Cumulative event-free survival curves were constructed using the Kaplan-Meier method, utilizing corresponding numbers-at-risk tables positioned beneath each plot to accurately account for the extensive follow-up period and progressive cohort attrition over time. Specifically, to characterize the unadjusted impact of baseline ageing on long-term outcomes, an age-stratified survival analysis was completed across the 40–49 (*n* = 373), 50–59 (*n* = 341), and ≥60-year (*n* = 126) cohorts. As visualized in *Figure 3*, the log-rank test demonstrated a highly significant separation in cumulative MACE-free survival across these distinct age strata (*P* < 0.0001), with the youngest cohort maintaining a consistently higher survival threshold throughout the 14-year tracking window.

**Table 6:** Distribution of cardiovascular outcomes according to atrial and ventricular ectopic burden (*n* = 840)

Outcome	Overall *n* = 149	Low AEB *n* = 41	High AEB *n* = 108	Low VEB *n* = 33	High VEB *n* = 116
ACS; *n* (%)	70 (46%)	21 (51%)	49 (45%)	17 (52%)	53 (46%)
CVA; *n* (%)	57 (38%)	15 (36%)	42 (39%)	15 (45%)	42 (36%)
Heart failure; *n* (%)	29 (19%)	7 (17%)	22 (20%)	3 (9%)	26 (22%)
CV-related death; *n* (%)	1 (0.6%)	0	0	0	1 (0.8%)

ACS, acute coronary syndrome; CVA, cerebrovascular accident; HF, heart failure; CV, cardiovascular; AEB, atrial ectopy burden; VEB, ventricular ectopic burden.

### Atrial ectopic burden and long-term outcomes

**Figure 4:**
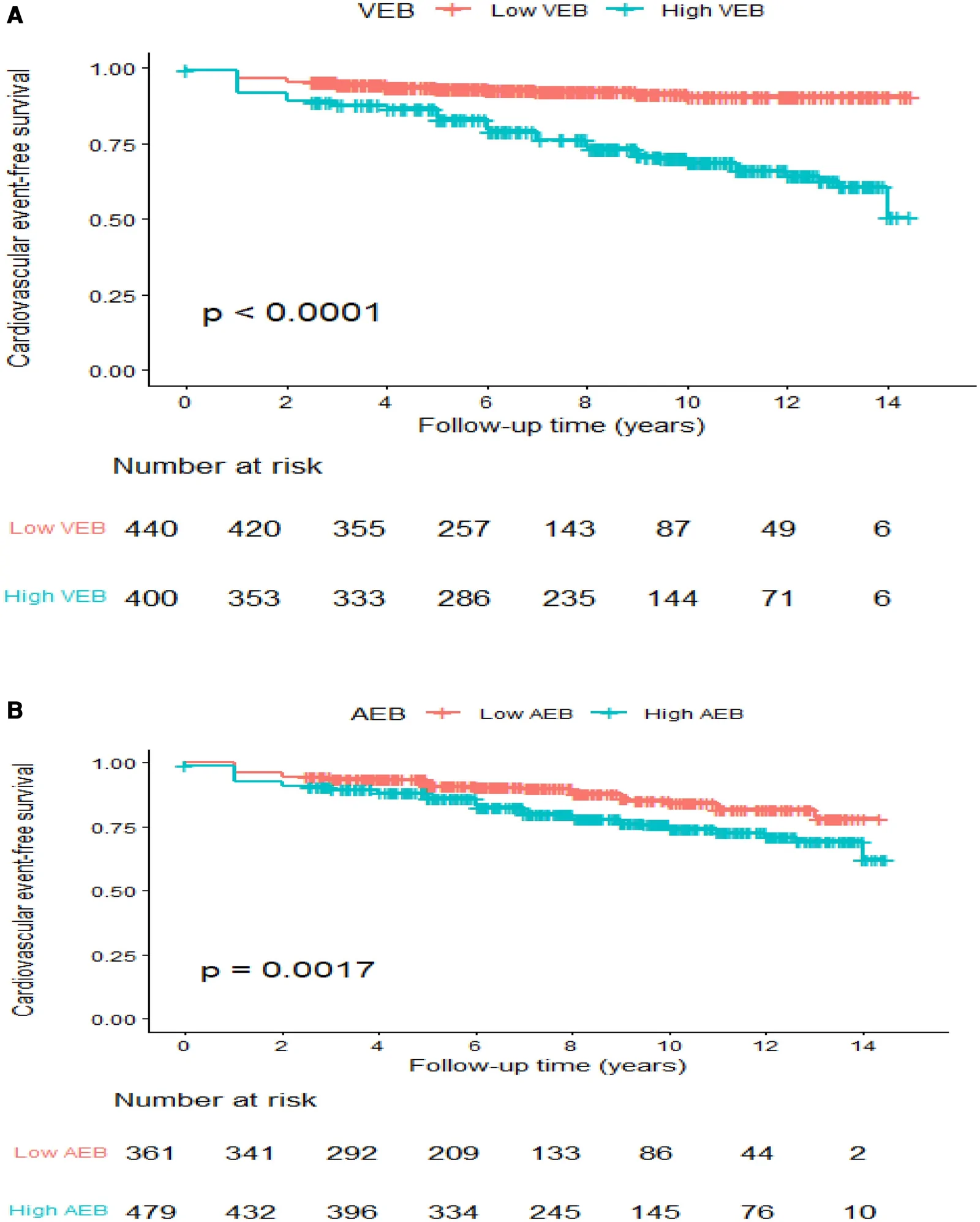
(*A*) Kaplan-Meier estimates of MACE-free survival stratified by ectopic burden. Cumulative MACE-free survival stratified by ventricular ectopic burden (high vs. low VEB), demonstrating a highly significant divergence over the 14-year follow-up window (log-rank *P* < 0.001). (*B*) Kaplan-Meier estimates of MACE-free survival stratified by ectopic burden. Cumulative MACE-free survival stratified by atrial ectopic burden (high vs. low AEB), illustrating the unadjusted separation that subsequently attenuates upon multivariable clinical adjustment. Corresponding numbers-at-risk tables are displayed beneath each panel. MACE, major adverse cardiovascular events; VEB, ventricular ectopic burden. AEB, atrial ectopic burden.

**Figure 5:**
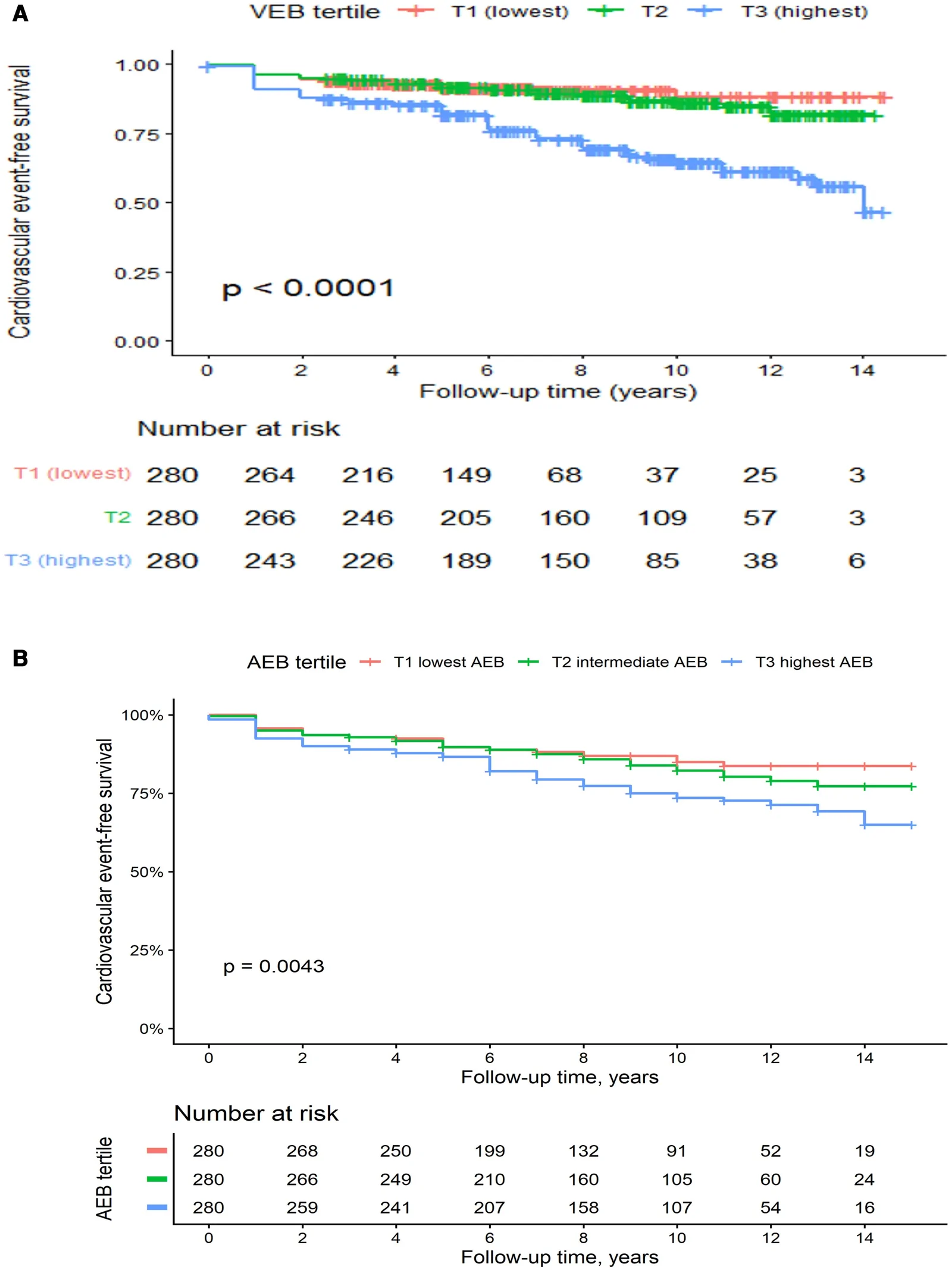
(*A*) Cardiovascular event-free survival by ventricular ectopy burden tertiles. Kaplan-Meier curves showing cardiovascular event-free survival stratified by tertiles of pure VEB, from T1 (lowest) to T3 (highest). Event-free survival differed significantly across VEB tertiles by log-rank test. In fully adjusted Cox regression including age, sex, beta-blocker use, and antiarrhythmic drug therapy, increasing VEB tertiles remained strongly associated with MACE. (*B*) Cardiovascular event-free survival by atrial ectopy burden tertiles. Cumulative survival curves stratified by tertiles of AEB, showing the unadjusted trend across escalating strata. Corresponding numbers-at-risk tables are displayed beneath each panel. MACE, major adverse cardiovascular events; VEB, ventricular ectopic burden. AEB, atrial ectopic burden.

Unadjusted Kaplan-Meier survival analysis demonstrated an initial separation in cumulative MACE-free survival between the low and high atrial ectopic burden groups (*Figure 4B*), as well as across escalating AEB tertiles (*Figure 5B*), likely reflecting baseline clinical confounding, particularly age. However, in multivariable Cox proportional hazards regression adjusted for age, sex, beta-blocker use, and antiarrhythmic drug therapy, high AEB was not independently associated with long-term adverse events (HR 1.30, 95% CI 0.89–1.90; *P* = 0.169; *Table 7*). Similarly, when pure AEB was evaluated across cohort-specific tertiles using the same fully adjusted model, increasing atrial ectopic burden showed only a borderline association with MACE that did not reach statistical significance (HR per tertile increase 1.21, 95% CI 0.98–1.50; *P* = 0.076). This borderline pattern was not reproduced in sensitivity analyses using quartiles (HR per group increase 1.11, 95% CI 0.95–1.29; *P* = 0.193) or quintiles (HR per group increase 1.07, 95% CI 0.95–1.20; *P* = 0.269).

**Table 7:** Medication-adjusted Cox regression for high atrial ectopic burden and cardiovascular events (*n* = 840)

Characteristic	HR	95% CI	*P* ^a^
Age (years)	1.03	1.00, 1.05	0.029
Male sex	3.24	1.42, 7.37	0.005
High AEB	1.30	0.89, 1.90	0.169
Beta-blocker use	2.05	1.46, 2.89	<0.001
Antiarrhythmic drug use	1.29	0.81, 2.06	0.287

AEB, atrial ectopic burden; CI, confidence interval; HR, hazard ratio.

^a^Cox regression model adjusted for age, sex, beta-blocker use, and antiarrhythmic drug use.

To isolate the prognostic impact of subclinical atrial ectopy from manifest atrial arrhythmia, a prespecified sensitivity analysis was conducted by excluding all individuals with baseline AF. AF was identified in 78 of 840 participants (9.3%). After excluding these patients, 762 participants remained, of whom 128 experienced cardiovascular events. High AEB was associated with a higher crude event rate (21.4% vs. 11.4%); however, this association was attenuated and was not statistically significant after adjustment for age, sex, beta-blocker use, and antiarrhythmic drug therapy (HR 1.32, 95% CI 0.90–1.95; *P* = 0.154). As an additional robustness check, we repeated the no-AF sensitivity analysis using a recalculated AEB median within the no-AF cohort; results were nearly identical, with high AEB remaining directionally associated with events but not statistically significant after adjustment (HR 1.33, 95% CI 0.89–1.98; *P* = 0.159).

### Ventricular ectopic burden and long-term outcomes

In stark contrast to the atrial findings, individuals with a high baseline VEB experienced a profound reduction in cumulative MACE-free survival compared to those with low ventricular ectopy (*Figure 4A*). Following rigorous multivariable adjustment for age, sex, baseline beta-blocker use, and antiarrhythmic medication exposure, high VEB remained a robust, independent predictor of long-term adverse clinical outcomes, conferring a three-fold increase in risk (HR 3.06, 95% CI 2.06–4.56; *P* < 0.001; *Table 8*). Furthermore, tertile-based trend analysis of pure VEB demonstrated a highly significant graded dose-response relationship, with each escalating VEB tertile associated with increased cardiovascular risk after adjustment for age, sex, beta-blocker use, and antiarrhythmic drug therapy (HR per tertile increase 2.02, 95% CI 1.60–2.53; *P* < 0.001) (*Figure 5A*). This graded association remained consistent in sensitivity analyses using quartiles (HR per group increase 1.70, 95% CI 1.44–2.00; *P* < 0.001) and quintiles (HR per group increase 1.46, 95% CI 1.28–1.65; *P* < 0.001).

**Table 8:** Medication-adjusted Cox regression for high ventricular ectopic burden and cardiovascular events (*n* = 840)

Characteristic	HR	95% CI	*P* ^a^
Age (years)	1.02	1.00, 1.05	0.064
Male Sex	3.30	1.45, 7.52	0.004
High VEB	3.06	2.06, 4.56	<0.001
Beta-blocker use	1.99	1.41, 2.80	<0.001
Antiarrhythmic drug use	1.15	0.72, 1.84	0.549

AEB, atrial ectopic burden; CI, confidence interval; HR, hazard ratio.

^a^Cox regression model adjusted for age, sex, beta-blocker use, and antiarrhythmic drug use.

### Temporal dynamics and serial Holter assessments

Serial Holter monitoring was available in 194 of 840 participants with longitudinal follow-up (23.1%). Among these individuals, 127 underwent two recordings, 31 underwent three recordings, and 36 underwent four or more recordings. The median interval between the first and last Holter recording was 3.91 years (IQR 2.08–6.13). However, repeat Holter recordings were clinically indicated rather than systematically scheduled according to a predefined study protocol. Therefore, to avoid immortal time bias and selection bias, only the earliest Holter recording was used for the primary analyses. Serial Holter findings were not incorporated into the primary survival models and are acknowledged as a limitation of the study design.

## Discussion

A central finding of this study is that atrial and ventricular ectopy, even at low absolute burdens, do not behave as equivalent markers of cardiovascular risk in asymptomatic adults without structural heart disease. While both AEB and VEB increase with advancing age, supporting a shared framework of age-related electrophysiological remodelling, their long-term prognostic trajectories diverge sharply upon multivariate adjustment. VEB remains a robust independent predictor of MACE after adjusting for age, sex, beta-blocker use, and antiarrhythmic drug use. Conversely, the crude survival association initially observed for AEB is entirely attenuated after accounting for these baseline medications, rendering the relationship statistically non-significant in the fully adjusted models. These findings may suggest a chamber-specific divergence in which ventricular ectopy may represent a stronger marker of clinically relevant electrical vulnerability, whereas atrial ectopy may be more sensitive to physiological and fitness-related factors.

The clinical utility of our cohort-specific threshold approach stems from the fact that conventional absolute cut-offs, such as a PVC burden >5–10%, were rarely met in this asymptomatic screening cohort; in this populations, relying strictly on high-burden thresholds fails to identify early or subtle electrophysiological vulnerability. Our data reveal that this low-burden phenotype is highly chamber-specific: the long-term prognostic signal is heavily concentrated in the ventricular substrate, whereas the atrial signal is attenuated by medication adjustment and the exclusion of baseline atrial fibrillation.

### Ectopy in the structurally normal heart

Population-based data have well established that ectopy increases with advancing age and can be frequently observed among individuals without overt cardiovascular disease. In the SAPALDIA cohort, atrial ectopy was nearly universal among adults aged ≥50 years, underscoring the powerful age-dependence of supraventricular ectopic activity,^13^ while the Copenhagen Holter Study linked excessive supraventricular ectopy to incident atrial fibrillation, stroke, and overall mortality.^14^ Our findings are consistent with these age-dependent patterns, yet they extend this paradigm to a low-risk population with no known structural heart disease.

### Chamber-specific divergence in determinants

A critical electrophysiological insight from this study is the stark chamber-specific divergence in clinical determinants between atrial and ventricular ectopy. In our expanded multivariable logistic regression model (*Table 4*), high AEB was independently driven by older age, reduced eGFR, male sex, and critically, low cardiorespiratory fitness. This supports the hypothesis that atrial ectopic vulnerability in asymptomatic adults is highly sensitive to systemic physiological stress, autonomic tone, and functional capacity. Conversely, traditional metabolic factors such as hypertension and obesity lacked a consistent independent contribution after multivariable adjustment,^11,15,16^ suggesting that in adults without structural heart disease, atrial ectopy reflects a highly dynamic, fitness-modulated substrate rather than advanced cardiometabolic pathology alone.

In contrast, the independent clinical drivers of ventricular ectopy proved entirely distinct. While prior literature from heterogeneous cohorts has associated VEB with modifiable risk factors like smoking or elevated blood pressure,^17–19^ our adjusted model (*Table 5*) shows that VEB is driven almost exclusively by older age and lower renal function. Notably, cardiorespiratory fitness demonstrated absolutely no independent association with VEB (*P* = 0.70). This complete divergence highlights a clear mechanistic divide: whereas atrial stability appears highly sensitive to aerobic capacity and physiological stress, as demonstrated by trials such as CARDIO-FIT,^20^ ventricular ectopy arises from an intrinsic myocardial ageing process and/or localized electrical heterogeneity that is fundamentally insulated from fitness levels. Taken together, these findings reinforce a model where atrial stability is functional, while ventricular stability is structural.

### Age-dependent changes in ectopic burden

The significant, graded increase in ectopic activity across age groups highlights the cellular mechanisms of electrophysiological ageing, such as progressive interstitial myocardial fibrosis, disrupted connexin-mediated cell-to-cell coupling, and heightened electrical heterogeneity.^10,21,22^ The profound, unadjusted impact of this chronological ageing process on long-term clinical outcomes is vividly illustrated by the stark survival separation observed across the age strata in *Figure 3* (log-rank *P* < 0.0001). Because chronological age remained a powerful independent predictor for both chambers within our multivariable regression models, ectopy serves as a sensitive biomarker of an ageing electrical substrate in otherwise normal hearts. Crucially, because VEB retained its strong, independent prognostic power for MACE even after forcing continuous age into the Cox models (*Table 8*), ventricular ectopy may capture subclinical myocardial risk that extends far beyond the raw chronological ageing gradients.

### Fitness and electrical stability

Cardiorespiratory fitness is a premier modifier of long-term survival, yet our data reveal that its protective capacity is highly chamber specific. Lower fitness was tightly coupled to high AEB (*Table 4*), matching data from the CARDIO-FIT trial, where improved aerobic performance directly reduced AF burden and optimized arrhythmia-free survival by mitigating autonomic fluctuations and systemic inflammation.^7,15,20,23^ In contrast, the absence of an independent association between fitness and VEB (*Table 5*) reflects the complex, multi-faceted relationship between physical activity and the ventricular substrate. While moderate physical activity optimizes global cardiovascular risk, extreme endurance training can induce structural right and left ventricular remodelling, resulting in a pro-arrhythmic phenotype in certain individuals.^24–27^ Our results might suggest that baseline aerobic capacity exerts a dominant, protective influence over the atrial chamber but does not alter the development of intrinsic ventricular ectopy. The exact volume and intensity of extreme physical activity could not be quantified in the current study; further research is required to see how competitive athletic training intersects with these relative burden thresholds.

### Impact on long-term outcomes

The prognostic implications of ectopic activity are highly context-dependent and are fundamentally shaped by the underlying myocardial substrate. In broadly healthy populations, isolated atrial or ventricular premature beats are often regarded as benign variants of normal ageing.^28,29^ However, specific arrhythmic patterns, most notably NSVT and complex atrial arrhythmias, have been linked to increased risks of mortality, stroke, and heart failure.^30,31^ Prior studies have largely focused on mixed-risk or disease-specific populations, where ectopy may reflect more advanced myocardial pathology.^32–34^ In contrast, over a median tracking period of 8 years within our follow-up, VEB emerged as a dominant, independent prognostic marker. High baseline VEB conferred a remarkable three-fold increase in the hazard of long-term MACE (HR 3.06, 95% CI 2.06–4.56; *Table 8*). Importantly, this ventricular prognostic signal was also evident when pure VEB was analysed across increasing burden categories, with a significant dose-response association observed across tertiles, quartiles, and quintiles. In contrast, pure AEB burden demonstrated only a borderline association in tertile analysis and was not consistent across alternative categorical thresholds. These findings reinforce a chamber-specific divergence in which ventricular ectopic burden provides a more robust and reproducible marker of long-term cardiovascular risk than atrial ectopic burden. This strongly indicates that high VEB can serve as an early warning sign of adverse myocardial risk before overt clinical disease manifests.

Conversely, the prognostic power of AEB proved highly sensitive to baseline clinical states and treatment choices. High AEB failed to preserve an independent relationship with MACE following medication adjustment (HR 1.30, *P* = 0.169; *Table 7*) and remained non-significant in our baseline atrial fibrillation exclusion sensitivity model (HR 1.32, *P* = 0.154). Ultimately, advanced ectopy in asymptomatic populations should not be viewed as an immediate therapeutic target for antiarrhythmic drugs or ablation, but rather as a crucial non-invasive biomarker. Identifying these divergent phenotypes can guide clinicians towards closer longitudinal surveillance and targeted primary prevention in individuals with elevated ventricular vulnerability.

### Limitations

This study has several limitations. First, its retrospective observational design precludes causal inference. Second, Holter monitoring was clinically indicated rather than performed universally, introducing possible selection and indication bias. Third, the primary analysis was based on baseline Holter recordings. Although 194 participants (23.1%) underwent repeat Holter monitoring, repeat testing was not systematically scheduled and was likely clinically driven; therefore, repeated-Holter analyses were considered exploratory, and ectopic burden was not modelled as a primary time-varying exposure. Fourth, beta-blocker and antiarrhythmic drug use were incorporated into the adjusted survival models, but data on medication dose, duration, adherence, and changes during follow-up were unavailable, leaving potential residual confounding by indication. Finally, the predominantly male tertiary screening cohort may limit generalizability, and occult subclinical myocardial disease cannot be fully excluded despite clinical evaluation and echocardiographic assessment in most participants.

## Conclusion

In a primary prevention population without known structural heart disease, atrial and ventricular ectopic burden increased with age and carried prognostic information. Ventricular ectopy emerged as the more robust independent predictor of long-term cardiovascular events, whereas atrial ectopy showed weaker and more adjustment-sensitive associations. Prospective studies with systematic serial rhythm monitoring are needed to clarify how ectopic burden should be incorporated into cardiovascular risk assessment.

## Supplementary Material

oeag133_Supplementary_Data

## Data Availability

The data underlying this article are not publicly available due to institutional and ethical restrictions but may be shared on reasonable request to the corresponding author.
