# Supplementary material for: Determinants and prognostic value of atrial and ventricular ectopic burden in adults without structural heart disease
Source: Eur Heart J Open. 2026 Aug 30;6(5):oeag133. doi: 10.1093/ehjopen/oeag133 (PMC13574498; doi:10.1093/ehjopen/oeag133)
Supplement: Supplementary file 1 [file oeag133_supplementary_data.docx]

| **Supplementary Table 1- Baseline Echocardiographic Characteristics of the Study Population** | | | | | | |
| --- | --- | --- | --- | --- | --- | --- |
| **Variable** | **N** |  | **Value** | | | |
| **LV end-diastolic diameter (mm)** | 751 |  | 4.9 (4.4-5) | | | |
| **Interventricular septal thickness (mm)** | 751 |  | 1.1 (0.9-1.1) | | | |
| **Posterior wall thickness (mm)** | 751 |  | 1.0 (0.9-1.0) | | | |
| **LV mass index (g/m²)** | 672 |  | 83 (72-96) | | | |
| **LV ejection fraction (%)** | 667 |  | | 60 ± 2.7 | |  |
| **E/A ratio**  **Mitral A velocity (cm/s)**  **LA diameter (cm)** | 629  629  627 |  | | 0.9 (0.7-1.7)  68 (57-80)  3.7 (3.4-4) | |  |
| **Pulmonary artery systolic pressure (mmHg)** | 627 |  | | 28 (25-31) | |  |
| Values are presented as mean ± standard deviation or median (interquartile range), according to data distribution. N indicates the number of participants with available measurements. Abbreviations: LV = left ventricular; LA = left atrial; SD = standard deviation; IQR = interquartile range; E/A = ratio of early (E) to late (A) diastolic mitral inflow velocities; PASP = pulmonary artery systolic pressure | | | | |  | |
